# Multimodal MRI-Based Radiomics-Clinical Model for Preoperatively Differentiating Concurrent Endometrial Carcinoma From Atypical Endometrial Hyperplasia

**DOI:** 10.3389/fonc.2022.887546

**Published:** 2022-05-27

**Authors:** Jieying Zhang, Qi Zhang, Tingting Wang, Yan Song, Xiaoduo Yu, Lizhi Xie, Yan Chen, Han Ouyang

**Affiliations:** ^1^ Department of Diagnostic Radiology, National Cancer Center/National Clinical Research Center for Cancer/Cancer Hospital, Chinese Academy of Medical Sciences and Peking Union Medical College, Beijing, China; ^2^ Department of Gynecologic Oncology, National Cancer Center/National Clinical Research Center for Cancer/Cancer Hospital, Chinese Academy of Medical Sciences and Peking Union Medical College, Beijing, China; ^3^ Department of Pathology, National Cancer Center/National Clinical Research Center for Cancer/Cancer Hospital, Chinese Academy of Medical Sciences and Peking Union Medical College, Beijing, China; ^4^ MR Research China, GE Healthcare, Beijing, China

**Keywords:** radiomics, magnetic resonance imaging, endometrial hyperplasia, endometrial neoplasms, texture analysis

## Abstract

**Objectives:**

To develop and validate a radiomics model based on multimodal MRI combining clinical information for preoperative distinguishing concurrent endometrial carcinoma (CEC) from atypical endometrial hyperplasia (AEH).

**Materials and Methods:**

A total of 122 patients (78 AEH and 44 CEC) who underwent preoperative MRI were enrolled in this retrospective study. Radiomics features were extracted based on T2-weighted imaging (T2WI), diffusion-weighted imaging (DWI), and apparent diffusion coefficient (ADC) maps. After feature reduction by minimum redundancy maximum relevance and least absolute shrinkage and selection operator algorithm, single-modal and multimodal radiomics signatures, clinical model, and radiomics-clinical model were constructed using logistic regression. Receiver operating characteristic (ROC) analysis, calibration curves, and decision curve analysis were used to assess the models.

**Results:**

The combined radiomics signature of T2WI, DWI, and ADC maps showed better discrimination ability than either alone. The radiomics-clinical model consisting of multimodal radiomics features, endometrial thickness >11mm, and nulliparity status achieved the highest area under the ROC curve (AUC) of 0.932 (95% confidential interval [CI]: 0.880-0.984), bootstrap corrected AUC of 0.922 in the training set, and AUC of 0.942 (95% CI: 0.852-1.000) in the validation set. Subgroup analysis further revealed that this model performed well for patients with preoperative endometrial biopsy consistent and inconsistent with postoperative pathologic data (consistent group, F1-score = 0.865; inconsistent group, F1-score = 0.900).

**Conclusions:**

The radiomics model, which incorporates multimodal MRI and clinical information, might be used to preoperatively differentiate CEC from AEH, especially for patients with under- or over-estimated preoperative endometrial biopsy.

## 1 Introduction

Atypical endometrial hyperplasia (AEH), also known as endometrial intraepithelial neoplasia, is considered a direct precursor of endometrial carcinoma (EC). Approximately 40% of AEH will proceed to EC within 12 months of onset (1, 2). In addition, previous studies have found that 37%-43% of AEH patients who undergo hysterectomy are diagnosed with concurrent endometrial carcinoma (CEC) on final pathology (3, 4).

Given the high risk of progression and CEC, the recommended treatment of AEH is total hysterectomy (with bilateral salpingo-oophorectomy when possible) in women who do not desire pregnancy. In contrast, non-surgical management may be appropriate for patients who plan on becoming pregnant in the future or those with comorbidities precluding surgical management (5). Previous studies have suggested that up to 12% of CEC patients suffer from high-grade tumors with deep myometrial invasion and have a 3-7% risk of lymph node involvement (6–9). Therefore, besides hysterectomy with bilateral salpingo-oophorectomy, a proportion of CEC patients may benefit from lymph node assessment as a guide to adjuvant therapy (10, 11). However, it is impossible to perform sentinel lymph node (SLN) mapping after hysterectomy due to disruption of the lymphatic channels originating from the uterine corpus and cervix during operation. Hence, an accurate preoperative diagnosis of AEH or CEC is crucial for selecting candidates for proper surgery or conservative treatment.

A primary diagnosis of AEH is usually made using dilation and curettage, hysteroscopy-guided biopsy, or hysteroscopic endometrial resection. Yet, these methods may fail to provide adequate tissue and lead to an improper diagnosis (12). Recent evidence suggested that non-invasive imaging tools may promote an accurate pre-treatment assessment of endometrial changes and optimize treatment planning (13). Magnetic resonance imaging (MRI) is a routine imaging modality used for the high-resolution evaluation of endometrial pathologies. Compared to conventional MRI, which has a relatively weak predictive value of CEC in patients with AEH (14, 15), the apparent diffusion coefficient (ADC) can be used to distinguish benign from malignant endometrial lesions (16). Still, so far, no studies have reported on the value of ADC in differentiating CEC from AEH.

Radiomics is a quantitative approach that extracts features from medical images using data-characterization algorithms and has been widely applied for differential diagnosis of cancers, evaluating therapeutic effects, and predicting the recurrence, metastasis, and survival time (17–19). A previous study used ^18^F-FDG PET/CT (positron emission tomography (PET) with 2-deoxy-2-[fluorine-18] fluoro-D-​glucose (18F-FDG)) quantitative parameters and texture analysis to distinguish CEC from AEH effectively (20). However, to the best of our knowledge, no research has determined whether an MRI-based radiomics study can detect CEC in AEH patients.

Thus, this study aimed to develop and validate a multimodal MRI-based radiomics-clinical model for detecting CEC from AEH before operation noninvasively. Also, we investigated the model performance in patients with preoperative endometrial biopsy consistent or inconsistent with postoperative pathological data.

## 2 Material and Methods

### 2.1 Patients

Our institutional ethics committee approved this study and waived the informed consent from patients. We retrospectively reviewed data of patients from our hospital database.

**Figure 1:**
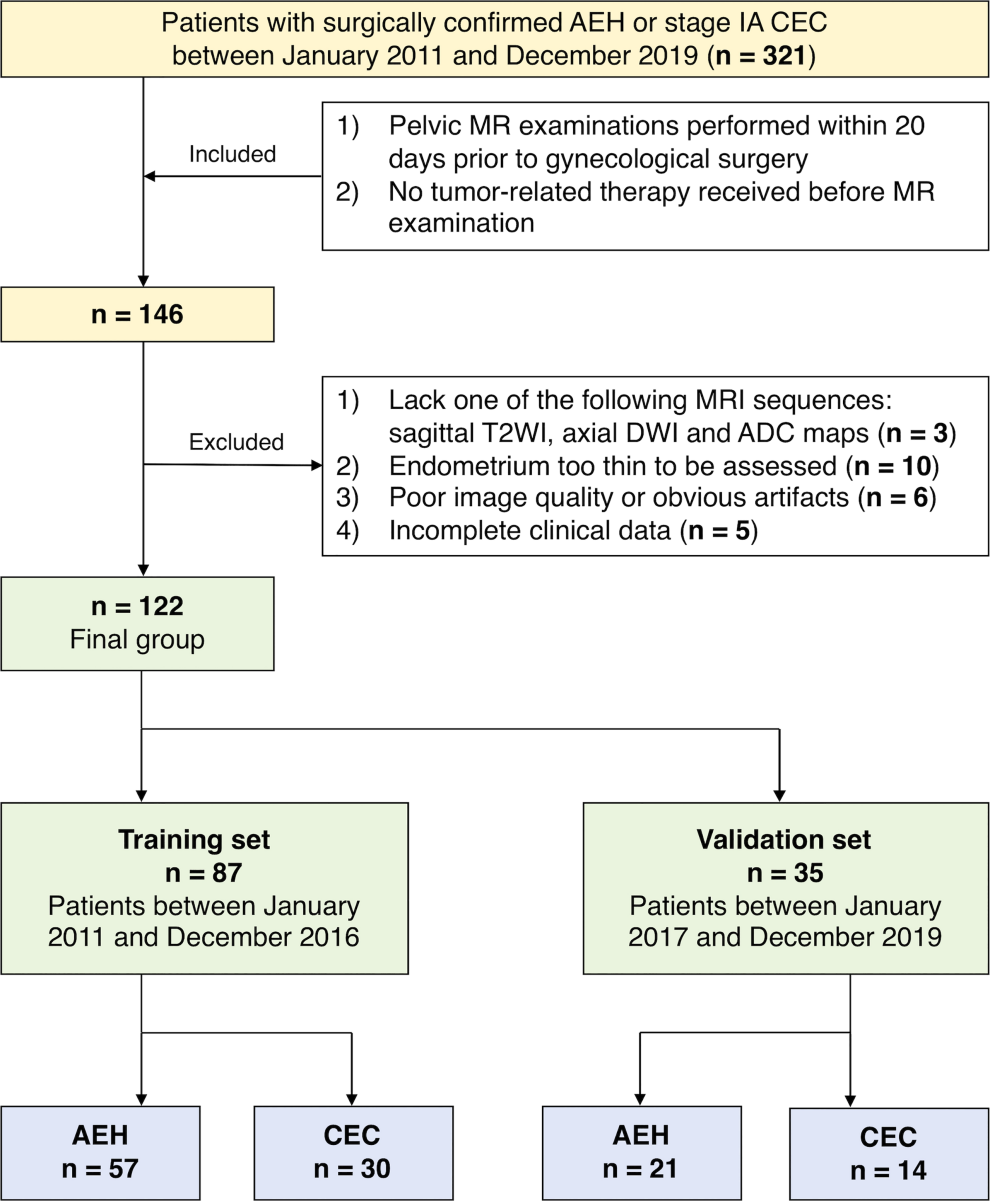
Flowchart of patient enrollment in this study.

In total, 321 patients who underwent gynecological surgery between January 2011 and December 2019 were pathologically confirmed with AEH or stage IA CEC. Inclusion criteria were: 1) AEH or stage IA CEC confirmed surgically and pathologically; 2) pelvic MRI performed within 20 days prior to gynecological surgery; 3) no tumor-related therapy received before MR examination. Exclusion criteria were the following: 1) lacking one of the following MRI sequences: sagittal T2-weighted imaging (T2WI), axial diffusion-weighted imaging (DWI), or the corresponding ADC map (n=3); 2) endometrium too thin (maximum thickness less than 4mm) to be assessed on MRI (n=10); 3) Poor image quality or obvious image artifacts affecting the visualization of tumor (n=6); 4) incomplete clinical data (n=5). Ultimately, MRI results of 122 patients (78 AEH and 44 CEC) were included in the study. The patients were divided into a training set (87 patients) and an independent validation set (35 patients) according to the time of treatment. A pathologist (Y.S.) with 20 years’ experience in gynecologic pathology reviewed the pathological data. **Figure 1** shows the flowchart of patient enrollment.

### 2.2 MRI Acquisition

All patients underwent conventional MR examination using 3.0T MR scanners (Signa HDxt and Discovery HD750, GE Medical System, Milwaukee, WI) with an eight-element phased-array wrap-around surface coil. Patients received an intramuscular injection of 10 mg raceanisodamine hydrochloride approximately 10 minutes before MRI to reduce bowel movement, excluding those with contraindications. The following sequences were included: sagittal T2WI and axial DWI. Diffusion gradients were applied in three orthogonal directions with *b* values of 0 and 800 s/mm^2^, and DWI with *b* value of 800 s/mm^2^ was involved in the analysis. ADC maps were manually generated from DWI on the post-processing workstation (Advantage Workstation 4.6; GE Medical System). Detailed sequence scanning parameters are shown in **Table 1**.

**Table 1:** Detailed Sequences Scanning Parameters in Two MR Scanners.

Parameters	Axial T1WI	Axial T2WI	SagittalT2WI	Axial oblique T2WI	AxialDWI	Axial T1WI postcontrast
**GE signa excite HD 3.0T**						
Technique	FSE	FS FSE	FSE	FSE	SS-EPI	3D LAVA-XV
TR (ms)/TE (ms)	620/8.2	5900/121	4920/139.1	4900/131.5	4400/64.3	4.1/1.8
FOV (cm)	38	34	30	22	34	35
Matrix (phase × frequency)	320×224	320×256	320×256	320×256	256×256	350×350
Slice thickness (mm)	5	5	4	3	5	1
Slice gap	1	1	0.4	0	1	0
Average (NEX)	2	2	2	4	2	1
b-value (s/mm^2^) ^*^	–	–	–	–	0, 800	–
**GE Discovery HD750 3.0T**						
Technique	LAVA-Flex	FS FSE	FSE	FSE	SS-EPI	3D LAVA-XV
TR (ms)/TE (ms)	4.2/1.3	4650/85.0	4220/125.4	5500/102.0	4000/56.1	7.9/4.1
FOV (cm)	38	34	30	22	34	35
Matrix (phase × frequency)	320×224	320×256	320×256	320×256	128×128	350×350
Slice thickness (mm)	3	5	4	3	5	1
Slice gap	0	1	0.4	0	1	0
Average (NEX)	1	2	2	4	2	1
b-value (s/mm^2^) ^*^	–	–	–	–	0, 800	–

*ADC maps were calculated voxel by voxel with the monoexponential model using the formula: ADC = In (S0/S800)/(b800−b0)

where S800 and S0 are the signal intensities with and without a diffusion gradient, respectively.

T1WI, T1-weighted imaging; T2WI, T2-weighted imaging; FS, fat suppression; FSE, fast-recovery fast spin-echo; DWI, diffusion-weighted imaging; SS-EPI, single-shot echo-planar imaging; LAVA-Flex, liver acquisition with volume acceleration; LAVA-XV, liver acquisition with volume acceleration-extended volume; TR, repetition time; TE, echo time; FOV, field of view; NEX, number of excitations.

### 2.3 Clinical and Conventional MR Assessment

The following clinical data were collected from medical records: age, body mass index, menopausal status, childbearing history, history of metabolic syndrome or polycystic ovary syndrome, history of endocrine therapy for breast cancer, blood serum cancer antigen 125 and cancer antigen 19-9 level, and preoperative pathological data.

Two radiologists (J.Z. and X.Y., with 6- and 18-years’ experience in gynecologic imaging, as Reader 1 and 2), who were blinded to the medical records and pathological data, independently measured endometrial stripe thickness on sagittal T2-weighted images. The average values were taken. Myometrial invasion [identified as interruption of the junction zone (21)] using all MR images was also assessed. The consistency between the two radiologists was evaluated by calculating Cohen’s kappa coefficients. Discrepancies were resolved by discussion until consensus was achieved.

### 2.4 Data Analysis

#### 2.4.1 Tumor Segmentation and Feature Extraction

Segmentation of images of the volume of interest (VOI) covering the whole tumor was performed using ITK-SNAP software (version 3.8.0, www.itksnap.org). AEH lesions would typically be presented with intermediate signal intensity on T2WI, DWI, and the ADC map compared with normal endometrium. Some endometrial lesions would be detected as CEC if the lesion was presented with isointense or slightly lower signal intensity on T2WI, higher signal intensity on DWI, and a lower value on ADC map compared with adjacent endometrium. In contrast, the remaining CEC lesions could not be delineated. Representative cases are presented in **Figure S1**. Each VOI was manually drawn along the contour of the entire endometrium or tumor (with visible tumor) slice-by-slice by Reader 1 on T2WI, DWI, and ADC map. Hemorrhagic, necrotic, cystic areas, and adjacent normal tissues were avoided using T1-weighted images and dynamic contrast-enhanced images as references. With a 1-month interval, the above procedure was repeated by Reader 1 and 2 independently. Each extracted feature’s inter- and intra-observer agreements were determined by calculating the intraclass correlation coefficients (ICC). Every case was then reviewed by another radiologist (H.O., with 30-years’ experience in gynecologic imaging) to ensure high-quality final segmentation results.

The feature extraction was realized using an open-source Python package called Pyradiomics (22). Before feature extraction, we applied image normalization in T2WI and DWI sequences using the Pyradiomics normalization method by centering it at the mean with standard deviation based on all gray values in the image (not just those inside the segmentation), thereby reducing the potential effects introduced by scanners, scanning parameters, and protocols. Then we applied Z score normalization to ensure that the radiomics features were measured on the same scale. The radiomics features were classified into three categories according to the feature calculation method: (1) 14 shape-based features; (2) 18 first-order statistical features; (3) 68 texture features, including gray level co-occurrence matrix, gray level size zone matrix (GLSZM), gray level run length matrix, and gray level dependence matrix (GLDM). Detailed radiomics features are listed in **Table S1**.

#### 2.4.2 Radiomic Feature Selection and Analysis

Stability analysis of radiomic features between inter-/intra- observer segmentations was first performed by removing the radiomic features with low reproducibility (ICC < 0.75). The remaining significant features were ranked using the minimum redundancy maximum relevance (mRMR) algorithm. Consequently, the top 10 features with low redundancy and high relevance were obtained for the following analyses.

**Figure 2:**
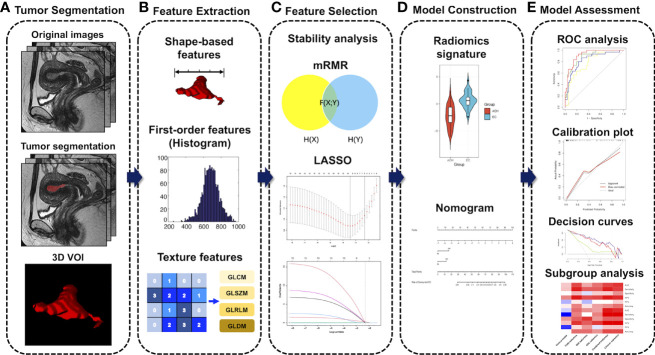
Workflow of radiomic analysis. **(A)** MR imaging segmentation. Three-dimensional (3D) segmentation of tumors in MR images. **(B)** Radiomic feature extraction. Radiomic features, including shape, intensity, and texture, were extracted from the tumor volume. **(C)** Feature selection process. The stability analysis, the minimum redundancy maximum relevance (mRMR), and the least absolute shrinkage and selection operator (LASSO) algorithm were used for the radiomic feature selection. **(D)** Model construction. Radiomics signatures were constructed using a binary logistic regression model. Finally, a nomogram for the optimal model was developed. **(E)** Model assessment. The performances of our models were evaluated by discrimination, calibration, and clinical utility, as well as subgroup analysis. VOI, volume of interest; GLCM, gray level co-occurrence matrix; GLSZM, gray level size zone matrix; GLRLM, gray level run length matrix; GLDM, gray level dependence matrix.

The least absolute shrinkage and selection operator (LASSO) algorithm was applied to avoid overfitting. The 1-standard error of the minimum criteria (the 1-SE criteria) was used to tune the regularization parameter (λ) and for feature selection using 10-fold cross-validation. T2WI, DWI, and ADC radiomics scores (T2WI-score, DWI-score, and ADC-score) were calculated for each patient using a weighted linear combination of selected features. Finally, a combined radiomics signature (Radscore) was generated using logistic regression based on T2WI, DWI, and ADC features. **Figure 2** shows the workflow of radiomic analysis.

#### 2.4.3 Clinical and Radiomics-Clinical Model Building, Discrimination, and Calibration

To select the optimal clinical parameters, the likelihood ratio test with Akaike’s information criterion was applied as the stopping rule for stepwise logistic regression analysis. The model with the lowest Akaike’s information criterion score was selected as a clinical model. Then, we developed a radiomics-clinical model based on Radscore and the optimal clinical parameters using multivariate logistic regression.

### 2.5 Statistical Analysis

Statistical analyses were performed using R software (version 4.0.3; http://www.Rproject.org). Differences between groups were assessed using t-tests or Mann-Whitney U tests for continuous variables; Chi-square test or Fisher’s exact test were applied for categorical variables. Receiver operating characteristic (ROC) curves were used to display and evaluate model performance. The area under the ROC curves (AUC), sensitivity, specificity, accuracy, and F1-score were used for evaluating the model performance. F1-score assumes that recall [equivalently, sensitivity, TP/(TP+FN)] and precision [equivalently, positive predictive value (PPV), TP/(TP+FP)] are of equal importance, where TP, FN, and FP represent true positive, false negative, and false positive, respectively. The higher F1-score synthetically reflects higher sensitivity and higher PPV. The formula for F1-score is as follows:


$$\text{F}1-\text{score }=\;\frac{2Precison\times Recall}{Precison+Recall}$$

DeLong’s test was used to compare the AUC of each model. Calibration curves and the Hosmer-Lemeshow test were used to assess the goodness of fit of the models. Decision curve analysis (DCA) was conducted to estimate the clinical usefulness of the models by calculating the net benefits at different values of threshold probability. Model internal validation in the training set was performed using the enhanced bootstrap resampling method (n=1000), which obtained the estimates of optimism in the regression models to provide a bias-corrected AUC value through a Somers’ D rank correlation metric whereby AUC = (1 + Somers’ D)/2. A *p* < 0.05 was considered statistically significant.

## 3 Results

### 3.1 Patient Characteristics

One hundred and twenty-two patients were enrolled in our study, including 78 AEH and 44 CEC patients. Baseline patients’ characteristics and preoperative biopsy results in the training and validation sets are summarized in **Table 2**.

**Table 2:** Baseline Characteristics of Patients in the Training and Validation sets.

Characteristics	Training Set (n=87)			Validation Set (n=35)			*p^#^* value
	AEH (n=57)	CEC (n=30)	*p* value	AEH (n=21)	CEC (n=14)	*p* value	
**Age, years, mean ± SD**	46.7 ± 4.9	46.7 ± 7.1	0.982	47.1 ± 5.2	48.2 ± 5.5	0.564	0.427
**BMI, kg/m2^†^**			0.610			0.697	0.752
≤24.9	26 (45.6)	11 (36.7)		12 (57.1)	7 (50.0)		
25~29.9	22 (38.6)	12 (40.0)		6 (28.6)	6 (42.9)		
≥30	9 (15.8)	7 (23.3)		3 (14.3)	1 (7.1)		
**Menopausal Status^†^**			0.377			0.721	0.148
Premenopausal	45 (78.9)	26 (86.7)		15 (71.4)	9 (64.3)		
Postmenopausal	12 (21.1)	4 (13.3)		6 (21.1)	4 (35.7)		
**Nulliparity^†^**	2 (3.5)	5 (16.7)	0.045^*^	1(4.8)	3 (21.4)	0.279	0.727
**CA125 (+)**	5 (8.8)	5 (16.7)	0.303	0 (0.0)	1 (7.1)	0.400	0.175
**CA19-9 (+)**	2 (3.5)	3 (10.0)	0.335	0 (0.0)	1 (7.1)	0.400	0.672
**Diabetes**	3 (5.3)	1 (3.3)	1.000	1 (4.8)	0 (0.0)	1.000	1.000
**PCOS**	0 (0.0)	1 (3.3)	0.345	0 (0.0)	1 (7.1)	0.400	0.493
**History of endocrine therapy^†^**	1 (1.8)	1 (3.3)	1.000	0 (0.0)	2 (14.3)	0.153	0.578
**Endometrial Thickness^†^**			0.005^*^			0.296	0.842
≤11mm	37 (64.9)	10 (33.3)		14 (66.7)	6 (42.9)		
>11mm	20 (35.1)	20 (66.7)		7 (33.3)	8 (57.1)		
**Myometrial invasion^†^**			0.126			0.685	0.295
No	51 (89.5)	23 (76.7)		17 (81.0%)	10 (71.4%)		
Yes	6 (10.5)	7 (23.3)		4(19.0%)	4(28.6%)		
**Preoperative Endometrial biopsy^†^**			<0.001^*^			0.002^*^	0.360
Hyperplasia without atypia	8 (14.0)	0 (0.0)		1 (4.8)	0 (0.0)		
Atypical hyperplasia	42 (73.7)	6 (20.0)		18 (85.7)	5 (35.7)		
Cancer	7 (12.3)	24 (80.0)		2 (9.5)	9 (64.3)		

^†^Data in parentheses are percentages.

*p < 0.05.

p^#^ value represents the comparison between training and validation sets.

AEH, atypical endometrial hyperplasia; CEC, concurrent endometrial carcinoma; BMI, body mass index; CA125, cancer antigen 125; CA19-9, cancer antigen 19-9; PCOS, polycystic ovary syndrome.

Based on the median, endometrial thickness (ET) was divided into ≤11mm and <11mm groups. Detailed information on ET in the subgroups (according to menopausal status and parity) of AEH and CEC patients is shown in **Table S2**. The consistency between the two radiologists was good to excellent in the evaluation of myometrial invasion (Kappa value=0.781) and measurement of ET (ICC = 0.908). In total, 29 (23.7%) patients (18 AEH and 11 CEC) had conflicting results between preoperative biopsy and postoperative pathology. Three (6.8%) patients in the CEC group had intermediate-risk EC (2 with non-endometrioid EC and 1 with high-grade tumor), and the remaining had low-risk EC, according to the 2021 ESGO/ESTRO/ESP guidelines for EC management (23).

### 3.2 Radiomics Signature Analysis

We extracted 300 features from the T2WI, DWI, and ADC maps of each VOI and reduced them to 283 by stability analysis. For T2WI, DWI, and ADC radiomics signature, the 7, 3, and 1 most relevant features were selected using the variable selection algorithm, respectively. Then, we determined the 5 top features consisting of 3 from T2WI, 1 from DWI, and 1 from ADC maps to build the combined radiomics signature (**Table 3**).

**Table 3:** Features of T2WI, DWI, ADC, and Combined Radiomics Signatures.

Feature Name	Coefficients
**T2WI Radiomics Signature**	
Intercept	-1.252
glszm_SizeZoneNonUniformityNormalized	-0.850
glszm_SmallAreaLowGrayLevelEmphasis	0.397
firstorder_10Percentile	0.054
shape_Maximum2DDiameterSlice	-0.871
shape_Flatness	0.769
firstorder_Skewness	1.100
gldm_LargeDependenceLowGrayLevelEmphasis	0.604
**DWI Radiomics Signature**	
Intercept	-0.777
shape_Maximum2DDiameterRow	-0.444
firstorder_Kurtosis	-0.740
shape_Flatness	0.678
**ADC Radiomics Signature**	
Intercept	-0.920
firstorder_10Percentile	-1.595
**Combined Radiomics Signature**	
Intercept	-1.235
T2WI_shape_Maximum2DDiameterSlice	-0.773
T2WI_gldm_LargeDependenceLowGrayLevelEmphasis	0.750
DWI_shape_Flatness	0.585
T2WI_firstorder_Skewness	-1.472
ADC_firstorder_10Percentile	0.529

**Figure 3:**
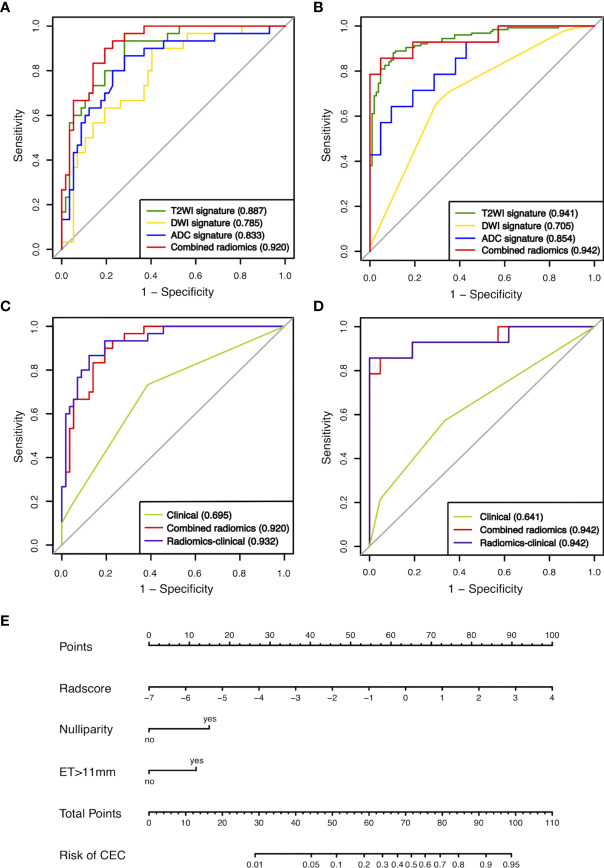
ROCs of the four radiomics signatures in the training **(A)** and validation **(B)** sets. ROCs of the clinical model, radiomics signature, and radiomics-clinical model in the training **(C)** and validation sets **(D)**. **(E)** Preoperative nomogram of the radiomics-clinical model. ET, endometrial thickness.

T2WI-score, DWI-score, ADC-score, and Radscore, calculated as the linear combination of these features with coefficients of the logistic regression model, were all significantly higher in the CEC group than the AEH group in the training set (all *p* < 0.001; **Figure S2**). The combined radiomics signature achieved the highest AUC of 0.920 and bootstrap corrected AUC of 0.892 in the training set and was then confirmed in the validation set with an AUC of 0.942 (**Table 4**). As shown in **Figures 3A, B**, Delong’s test demonstrated statistical differences in AUC values between the combined and DWI radiomics signature (*p* = 0.030) in the validation set. The mathematical formula used to calculate radiomics scores is shown in **Method S1**.

**Table 4:** Performances of Different Models in the Training and Validation Sets.

Model	Data sets	AUC	95%CI	Bootstrap Corrected AUC	Sensitivity	Specificity	Accuracy	F1-score
T2WI Radiomics	Training Set	0.887	0.818-0.956	0.838	0.930	0.720	0.790	0.843
	Validation Set	0.895	0.778-1.000	NA	0.929	0.857	0.886	0.897
DWI Radiomics	Training Set	0.785	0.688-0.883	0.752	0.900	0.600	0.700	0.781
	Validation Set	0.735	0.566-0.903	NA	0.500	0.904	0.743	0.627
ADC Radiomics	Training Set	0.833	0.741-0.925	0.832	0.870	0.720	0.770	0.807
	Validation Set	0.854	0.729-0.979	NA	0.643	0.905	0.800	0.739
Combined Radiomics	Training Set	0.920	0.865-0.974	0.892	0.900	0.810	0.840	0.860
	Validation Set	0.942	0.857-1.000	NA	0.857	0.952	0.914	0.900
Clinical Model	Training Set	0.708	0.588-0.827	0.687	0.730	0.670	0.690	0.692
	Validation Set	0.641	0.448-0.834	NA	0.571	0.667	0.629	0.600
Clinical-Radiomics Model	Training Set	0.932	0.880-0.984	0.922	0.870	0.880	0.870	0.871
	Validation Set	0.942	0.852-1.000	NA	0.857	1.000	0.943	0.923

ACC, accuracy; AUC, area under the receiver operating characteristic curve; CI, confidence interval; NA, not applicable.

### 3.3 Clinical and Radiomics-Clinical Model Construction and Performance Assessment

In the clinical model, two parameters were independently associated with CEC in AEH patients, including the status of nulliparity (odds ratio [OR]: 7.082; 95% confidence interval [CI]: 1.159-43.288; p = 0.034) and ET>11mm (OR: 4.148, 95%CI: 1.553-11.073; p = 0.005). These two parameters, along with Radscore, were used to build the radiomics-clinical model. Nomogram (**Figure 3E**) was established for this model. The auto- and cross-correlations of selected features in the radiomics-clinical model derived from the training set are shown in **Figure S3**.

**Figure 4:**
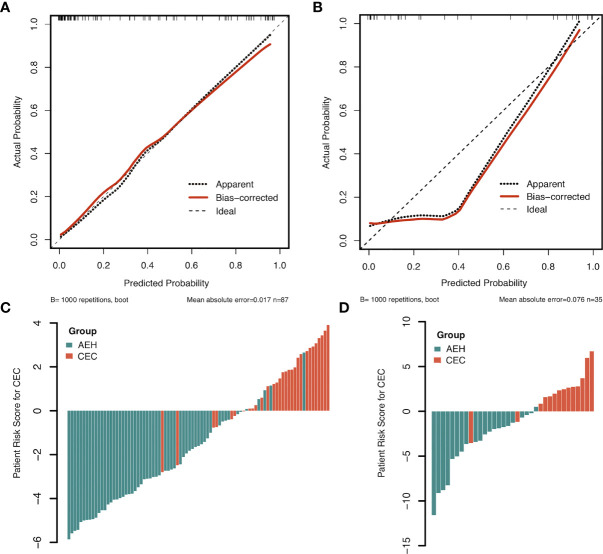
The calibration plots of the radiomics-clinical model in the training **(A)** and validation sets **(B)**. Patient risk scores output by the radiomics-clinical model in the training **(C)** and validation sets **(D)**, while orange bars show scores for those who have concurrent endometrial carcinoma.

**Figure 5:**
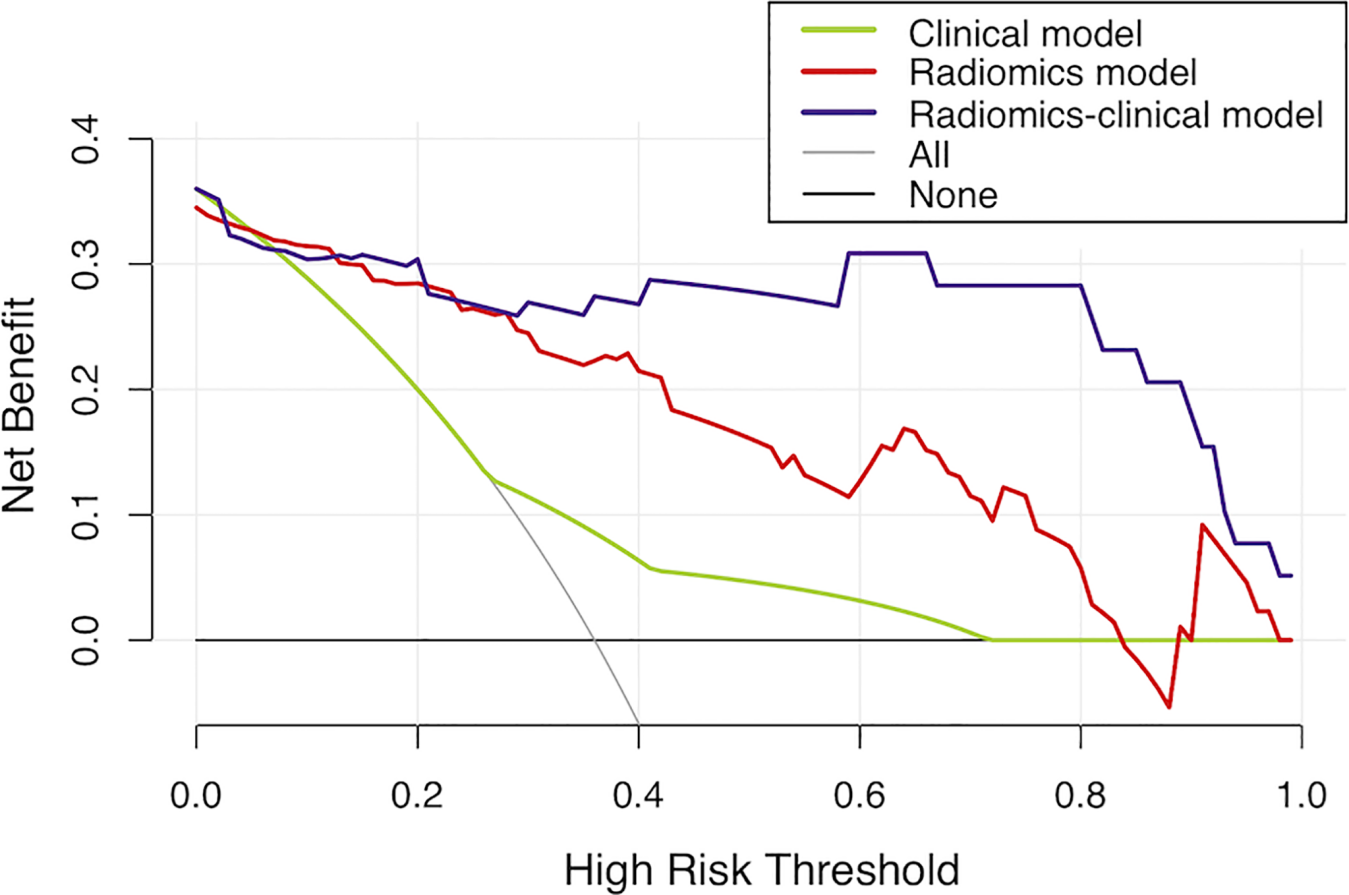
Decision curve analysis for the models in the validation set. It can be concluded that when the threshold probability is over 30% approximately, the radiomics-clinical model could provide extra profits over the “treat-all” or “treat-none” scheme, the combined radiomics signature, and the clinical model.

The clinical model showed moderate performance with AUC of 0.695 and 0.641, which was significantly improved by the radiomics-clinical model to 0.932 and 0.942 in the training and validation sets, respectively (Delong’s test, *p* < 0.001; **Figures 3C, D**). There was no significant difference between the AUC of the combined radiomics signature and radiomics-clinical model in the training and validation sets (Delong’s test, *p*<0.05). Calibration curves showed good fitness for the radiomics-clinical model (Hosmer-Lemeshow test, *p* = 0.933 in the training set, 0.400 in the validation sets) (**Figures 4A, B**). The patients’ risk scores, indicating the models’ high classification ability, are shown in **Figures 4C, D**. DCA of the models is shown in **Figure 5**.

**Figure 6:**
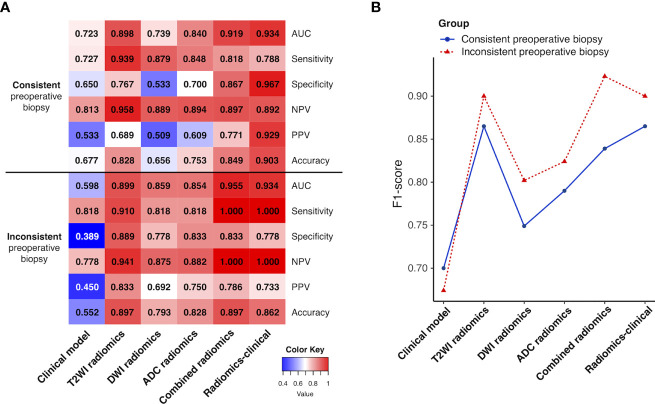
**(A)** Heatmap showing the models’ performance in the subgroups of patients with preoperative endometrial biopsy consistent and inconsistent with postoperative pathologic data. A deeper red indicates a larger value. **(B)** Line chart of the F1-score of the models in two subgroups. NPV, negative predictive value; PPV, positive predictive value.

As shown in **Figure 6A**, in the subgroup of patients with preoperative endometrial biopsy inconsistent (under- or over-estimated) with postoperative pathology, the combined radiomics signature and radiomics-clinical model achieved the highest sensitivity and NPV of 1.000, with an AUC of 0.955 and 0.934, respectively. The F1-score of the two subgroups is shown in **Figure 6B**. The radiomics-clinical model showed good potency among the six classification models (consistent group, F1-score = 0.865; inconsistent group, F1-score = 0.900), while the combined radiomics signature performed even better in patients with inconsistent biopsy results (F1-score = 0.923).

## 4 Discussion

In this study, we developed a multimodal MRI-based radiomics-clinical model for preoperative differentiation of CEC from AEH. The model consisting of radiomics features and clinical data (ET >11mm and nulliparity status) demonstrated the best discrimination ability and goodness of fit. Moreover, in patients with under- or over-estimated preoperative biopsy results, the sensitivity and NPV were greatly improved after applying the model with relatively high PPV. Furthermore, despite differences in the MR scanners among various subjects, the radiomics-clinical model revealed an excellent capacity for detecting CEC from AEH in the internal validation, with a bootstrap corrected AUC of 0.922 in the training set and AUC of 0.942 in the validation set, thus surpassing other models.

Previous studies (24–27) showed that AEH and EC shared common predisposing risk factors, such as age, postmenopausal status, nulliparity, obesity, diabetes, PCOS, and long-term tamoxifen therapy. Liakou et al. (14) found that myometrial invasion on conventional MRI was associated with increased CEC risk for AEH patients; nevertheless, the sensitivity and specificity of MRI in identifying cancer were poor (37% and 89%, respectively). In the current study, we adopted the aforementioned clinical parameters into our clinical predictive model. Nulliparity and ET >11mm observed on conventional MRI were found to be independently associated with the differentiation of CEC from AEH. Nulliparity is an established risk factor for endometrial cancer, and each pregnancy provides an additional risk reduction (28). The study of ET as a predictive factor for endometrial pathology with abnormal uterine bleeding is a debated topic with conflicting results, especially in premenopausal patients, since its predictive performance is affected by menstrual cycle phases. Vetter et al. (29) demonstrated that ET >2cm on preoperative transvaginal ultrasound was associated with increased odds of CEC in AEH patients while controlling for age. Wise et al. (30) proved a strong association between ET > 11 mm and AEH/EC in premenopausal women. Based on the median, we found that the same ET cut-off value (>11 mm) on MRI was associated with CEC in AEH patients in the current study. Moreover, our study produced consistent results that a higher proportion of CEC than AEH patients had an ET >11mm in different subgroups based on menopausal status and parity. However, the clinical model’s performance was unsatisfactory, especially for patients with inconsistent preoperative biopsy results.

Next, we constructed radiomics signatures based on different MRI images (T2WI, DWI, and ADC maps). T2WI radiomics signature performed better for categorizing CEC and AEH than DWI. A reasonable explanation could be that T2WI is the critical conventional sequence of non-enhanced MRI in diagnosing endometrial diseases, providing detailed anatomical characteristics with high contrast and spatial resolution. On T2WI, AEH usually has a similar signal intensity with that of the normal endometrium, while EC shows intermediate-low signal intensity relative to hyperintense normal endometrium (21, 31). In this study, multiple T2WI radiomic features were selected in the T2WI radiomics signature, such as tumor shape, intensity, and gray level texture features (from GLSZM and GLDM), reflecting different aspects of intratumor heterogeneity and thus improving the discriminative ability of CEC and AEH.

CEC patients tend to have small tumors that may not be associated with endometrial thickening or have a signal intensity similar to that of the normal endometrium. In those cases, functional sequences, such as DWI, can be beneficial. A high b-value makes images more sensitive to water diffusion, thus increasing contrast enhancement between normal and cancerous tissue (32). Therefore, the presence of restricted diffusion on DWI within thickened endometrium will raise suspicion for the existence of EC. This study included only first-order statistics (Kurtosis) and shape-based features (Maximum 2D Diameter Row, Flatness) in the DWI radiomics signature. No other texture features were highly correlated to the classification task, probably due to its relatively poor spatial resolution. Flatness was included in both T2WI and DWI radiomics signatures, disillusioning largest from smallest principal components in the VOI shape, with a value range between 1 (non-flat, sphere-like) and 0 (a flat object, or single-slice segmentation). Flatness may provide information as complementation for ET in detecting small-size CEC from AEH.

Numerous studies have reported that ADC measurements (without confounding T1 or T2 effects of DWI signal) could be used as additional tools for differentiating between benign and malignant conditions (19, 33, 34). Moharamzad et al. (35) performed a meta-analysis and concluded that the combined sensitivity and specificity of mean ADC values for differentiating EC from benign lesions were 93% and 94%, respectively. Chen et al. (36) developed an MRI-based radiomics model including ADC_10Percentile for distinguishing EC from its benign mimics. Furthermore, Yan and colleagues (37) selected ADC_10Percentile as the only component of ADC radiomics signature in developing a radiomic nomogram predicting high-risk EC preoperatively. Similarly, we found that ADC_10Percentile may further promote the differentiation of CEC from AEH, compared to mean ADC values. A possible explanation is that lower percentiles of ADC may better represent aggressive solid components within CEC (38).

Finally, we discovered that a combined radiomics signature and radiomics-clinical model obtained more precise and comprehensive information about the tumors and yielded better diagnostic performance in the classification tasks than single-modal signatures. In clinical practice, it commonly happens that endometrial sampling is not possible (usually due to cervical stenosis) or the histopathology results are inconclusive or inconsistent with the clinical suspicion. Our study proved that the combined radiomics signature and radiomics-clinical models performed fairly well, especially for patients with preoperative endometrial biopsy inconsistent with postoperative pathologic data, thus indicating the supplementary value of MRI-based radiomics to preoperative endometrial biopsy.

Accurate preoperative prediction of the presence of CEC in AEH patients is vital for making proper personalized treatment decisions and assessing the prognosis of patients. Previous studies exploring the risk of CEC in patients with AEH have mainly focused on examining clinical factors such as sampling methods and histologic characteristics of AEH (39, 40). For the first time, we have developed and validated a multimodal MRI-based radiomics-clinical model for evaluating tumor heterogeneity and thus detecting CEC from AEH preoperatively. Strengths of this study include final pathology review at a single institution and the inclusion of clinical data as well as objective quantitative parameter (Radscore) to better predict the risk of underlying cancer at the time of hysterectomy for AEH. Knowledge of lymph node status in EC patients would allow a more tailored recommendation for postoperative therapy or surveillance. More recently, SLN mapping has been introduced into the surgical management of EC to obtain adequate nodal status information with a reduction in lymphadenectomy-related morbidity (such as lymphedema and lymphocele) (41). It is essential to know that the ability to perform SLN mapping in EC depends on intact lymphatic channels, and it cannot be performed after hysterectomy (29). AEH patients diagnosed with high-risk EC at the time of hysterectomy alone would then subsequently require a full lymphadenectomy. Therefore, for AEH patients with a high risk of CEC evaluated by our preoperative radiomics-clinical model, SLN mapping during hysterectomy should be considered.

The present study has some limitations. First, this was a retrospective study conducted at a single center and with a relatively small sample size. We have to acknowledge that despite the results of this study being promising, further investigation with larger study cohorts is necessary to validate our preliminary study. Second, AEH could not be accurately contoured with similar signal intensity to normal endometrium, while some CEC lesions could be detected on multimodal MR images (we contoured the visible tumor as VOIs in these cases). The bias introduced by inconsistency in VOI drawing was inevitable; however, it reflected the “real world” of routine diagnostic work. It was minimized by consulting another experienced radiologist in our study. Third, although we excluded patients with ET<4mm in this study because of the limitation of visual evaluation, the risk of developing CEC was relatively low in both pre- and post-menopausal women (42, 43). Finally, genomic information was not yet obtained and incorporated into our models. A combination of gene marker panels and radiomic features could have an extraordinary impact on the management of AEH in future studies.

To sum up, this new diagnostic model incorporating multimodal MRI-based radiomics and clinical information may be used to distinguish CEC from AEH noninvasively and effectively before the operation, especially for patients with under- or over-estimated preoperative endometrial biopsy. Nevertheless, a multicenter study with a larger dataset is needed to further validate our models’ reproducibility and generalizability.

## Data Availability Statement

The raw data supporting the conclusions of this article will be made available by the authors, without undue reservation.

## Ethics Statement 

The studies involving human participants were reviewed and approved by the ethics committee of National Cancer Center/National Clinical Research Center for Cancer/Cancer Hospital, Chinese Academy of Medical Sciences and Peking Union Medical College. Written informed consent for participation was not required for this study in accordance with the national legislation and the institutional requirements.

## Author Contributions

JZ: conceptualization, formal analysis, validation, visualization, funding acquisition, writing - original draft, writing - review & editing. QZ: investigation, data curation, software. TW: conceptualization, investigation, data curation. YS: investigation, resources. XY: conceptualization, methodology, formal analysis, writing - review & editing. LX: resources, software. YC: project administration. HO: project administration, supervision. All authors contributed to the article and approved the submitted version.

## Funding

This work was supported by the Special Research Fund for Central Universities, Peking Union Medical College (No. 3332021032).

## Conflict of Interest

Author LX was employed by GE Healthcare.

The remaining authors declare that the research was conducted in the absence of any commercial or financial relationships that could be construed as a potential conflict of interest.

## Publisher’s Note

All claims expressed in this article are solely those of the authors and do not necessarily represent those of their affiliated organizations, or those of the publisher, the editors and the reviewers. Any product that may be evaluated in this article, or claim that may be made by its manufacturer, is not guaranteed or endorsed by the publisher.

## Abbreviations

AEH, Atypical endometrial hyperplasia; ADC, Apparent diffusion coefficient; AUC, Area under the receiver operating characteristic curve; CEC, Concurrent endometrial carcinoma; DWI, Diffusion-weighted imaging; EC, Endometrial carcinoma; ET, Endometrial thickness; GLDM, Gray level dependence matrix; GLSZM, Gray level size zone matrix; ICC, Intraclass correlation coefficients; LASSO, Least absolute shrinkage and selection operator; MRI, Magnetic resonance imaging; mRMR, Minimum redundancy maximum relevance; NPV, Negative predictive value; PPV, Positive predictive value; SLN, Sentinel lymph node; ROC, Receiver operating characteristic curve; T2WI, T2-weighted imaging; VOI, Volume of interest.

## References

[1] Mutter GL Kauderer J Baak JP Alberts D. Biopsy Histomorphometry Predicts Uterine Myoinvasion by Endometrial Carcinoma: A Gynecologic Oncology Group Study. Hum Pathol (2008) 39(6):866–74. doi: 10.1016/j.humpath.2007.09.023 PMC2601480 18436277

[2] Baak JP Mutter GL Robboy S van Diest PJ Uyterlinde AM Orbo A. The Molecular Genetics and Morphometry-Based Endometrial Intraepithelial Neoplasia Classification System Predicts Disease Progression in Endometrial Hyperplasia More Accurately Than the 1994 World Health Organization Classification System. Cancer (2005) 103(11):2304–12. doi: 10.1002/cncr.21058 PMC2600877 15856484

[3] Trimble CL Kauderer J Zaino R Silverberg S Lim PC Burke JJ 2nd. Concurrent Endometrial Carcinoma in Women With a Biopsy Diagnosis of Atypical Endometrial Hyperplasia: A Gynecologic Oncology Group Study. Cancer (2006) 106(4):812–9. doi: 10.1002/cncr.21650 16400639

[4] Rakha E Wong SC Soomro I Chaudry Z Sharma A Deen S. Clinical Outcome of Atypical Endometrial Hyperplasia Diagnosed on an Endometrial Biopsy: Institutional Experience and Review of Literature. Am J Surg Pathol (2012) 36(11):1683–90. doi: 10.1097/PAS.0b013e31825dd4ff 23073327

[5] Auclair MH Yong PJ Salvador S Thurston J Colgan TTJ Sebastianelli A. Guideline No. 392-Classification and Management of Endometrial Hyperplasia. J Obstet Gynaecol Can (2019) 41(12):1789–800. doi: 10.1016/j.jogc.2019.03.025 31785798

[6] Karamursel BS Guven S Tulunay G Kucukali T Ayhan A. Which Surgical Procedure for Patients With Atypical Endometrial Hyperplasia? Int J Gynecol Cancer (2005) 15(1):127–31. doi: 10.1111/j.1048-891X.2005.15013.x 15670307

[7] Touhami O Grégoire J Renaud MC Sebastianelli A Grondin K Plante M. The Utility of Sentinel Lymph Node Mapping in the Management of Endometrial Atypical Hyperplasia. Gynecol Oncol (2018) 148(3):485–90. doi: 10.1016/j.ygyno.2017.12.026 29290489

[8] Whyte JS Gurney EP Curtin JP Blank SV. Lymph Node Dissection in the Surgical Management of Atypical Endometrial Hyperplasia. Am J Obstet Gynecol (2010) 202(2):176.e1–.e4. doi: 10.1016/j.ajog.2009.10.855 20022313

[9] Costales AB Schmeler KM Broaddus R Soliman PT Westin SN Ramirez PT. Clinically Significant Endometrial Cancer Risk Following a Diagnosis of Complex Atypical Hyperplasia. Gynecol Oncol (2014) 135(3):451–4. doi: 10.1016/j.ygyno.2014.10.008 PMC4268403 25316176

[10] Colombo N Creutzberg C Amant F Bosse T González-Martín A Ledermann J. ESMO-ESGO-ESTRO Consensus Conference on Endometrial Cancer: Diagnosis, Treatment and Follow-Up. Int J Gynecol Cancer (2016) 26(1):2–30. doi: 10.1097/igc.0000000000000609 26645990 PMC4679344

[11] Jang JW Lee LJ. External Beam, Brachytherapy, or Chemotherapy? Defining Adjuvant Therapy for Early-Stage and High- and High-Intermediate-Risk Endometrial Cancer. J Clin Oncol (2019) 37(21):1778–84. doi: 10.1200/jco.19.00362 31163010

[12] Clark TJ Voit D Gupta JK Hyde C Song F Khan KS. Accuracy of Hysteroscopy in the Diagnosis of Endometrial Cancer and Hyperplasia: A Systematic Quantitative Review. JAMA (2002) 288(13):1610–21. doi: 10.1001/jama.288.13.1610 12350192

[13] Haldorsen IS Salvesen HB. What Is the Best Preoperative Imaging for Endometrial Cancer? Curr Oncol Rep (2016) 18(4):25. doi: 10.1007/s11912-016-0506-0 26922331 PMC4769723

[14] Liakou CG La Russa MC Akrivos N Ames V Scott-Barrett S Duncan TJ. The Role of Magnetic Resonance Imaging in the Pre-Operative Evaluation of Women Diagnosed With Atypical Endometrial Hyperplasia. Anticancer Res (2020) 40(5):2989–93. doi: 10.21873/anticanres.14279 32366453

[15] Natarajan P Vinturache A Hutson R Nugent D Broadhead T. The Value of MRI in Management of Endometrial Hyperplasia With Atypia. World J Surg Oncol (2020) 18(1):34. doi: 10.1186/s12957-020-1811-5 32041614 PMC7011375

[16] Fujii S Matsusue E Kigawa J Sato S Kanasaki Y Nakanishi J. Diagnostic Accuracy of the Apparent Diffusion Coefficient in Differentiating Benign From Malignant Uterine Endometrial Cavity Lesions: Initial Results. Eur Radiol (2008) 18(2):384–9. doi: 10.1007/s00330-007-0769-9 17917730

[17] Zhao L Gong J Xi Y Xu M Li C Kang X. MRI-Based Radiomics Nomogram May Predict the Response to Induction Chemotherapy and Survival in Locally Advanced Nasopharyngeal Carcinoma. Eur Radiol (2020) 30(1):537–46. doi: 10.1007/s00330-019-06211-x 31372781

[18] Wormald BW Doran SJ Ind TE D'Arcy J Petts J deSouza NM. Radiomic Features of Cervical Cancer on T2-And Diffusion-Weighted MRI: Prognostic Value in Low-Volume Tumors Suitable for Trachelectomy. Gynecol Oncol (2020) 156(1):107–14. doi: 10.1016/j.ygyno.2019.10.010 PMC7001101 31685232

[19] Xie H Hu J Zhang X Ma S Liu Y Wang X. Preliminary Utilization of Radiomics in Differentiating Uterine Sarcoma From Atypical Leiomyoma: Comparison on Diagnostic Efficacy of MRI Features and Radiomic Features. Eur J Radiol (2019) 115:39–45. doi: 10.1016/j.ejrad.2019.04.004 31084757

[20] Wang T Sun H Guo Y Zou L. (18)F-FDG PET/CT Quantitative Parameters and Texture Analysis Effectively Differentiate Endometrial Precancerous Lesion and Early-Stage Carcinoma. Mol Imaging (2019) 18:1–10. doi: 10.1177/1536012119856965 PMC6572902 31198089

[21] Takeuchi M Matsuzaki K Uehara H Yoshida S Nishitani H Shimazu H. Pathologies of the Uterine Endometrial Cavity: Usual and Unusual Manifestations and Pitfalls on Magnetic Resonance Imaging. Eur Radiol (2005) 15(11):2244–55. doi: 10.1007/s00330-005-2814-x 16228215

[22] van Griethuysen JJM Fedorov A Parmar C Hosny A Aucoin N Narayan V. Computational Radiomics System to Decode the Radiographic Phenotype. Cancer Res (2017) 77(21):e104–e7. doi: 10.1158/0008-5472.CAN-17-0339 PMC5672828 29092951

[23] Concin N Matias-Guiu X Vergote I Cibula D Mirza MR Marnitz S. ESGO/ESTRO/ESP Guidelines for the Management of Patients With Endometrial Carcinoma. Int J Gynecol Cancer (2021) 31(1):12–39. doi: 10.1136/ijgc-2020-002230 33397713

[24] Wise MR Jordan V Lagas A Showell M Wong N Lensen S. Obesity and Endometrial Hyperplasia and Cancer in Premenopausal Women: A Systematic Review. Am J Obstet Gynecol (2016) 214(6):689–97. doi: 10.1016/j.ajog.2016.01.175 26829507

[25] Giannella L Cerami LB Setti T Bergamini E Boselli F. Prediction of Endometrial Hyperplasia and Cancer Among Premenopausal Women With Abnormal Uterine Bleeding. BioMed Res Int (2019) 2019:8598152. doi: 10.1155/2019/8598152 31011581 PMC6442314

[26] Weber AM Belinson JL Piedmonte MR. Risk Factors for Endometrial Hyperplasia and Cancer Among Women With Abnormal Bleeding. Obstet Gynecol (1999) 93(4):594–8. doi: 10.1016/s0029-7844(98)00469-4 10214840

[27] Sanderson PA Critchley HO Williams AR Arends MJ Saunders PT. New Concepts for an Old Problem: The Diagnosis of Endometrial Hyperplasia. Hum Reprod Update (2017) 23(2):232–54. doi: 10.1093/humupd/dmw042 PMC5850217 27920066

[28] Troisi R Bjorge T Gissler M Grotmol T Kitahara CM Myrtveit Saether SM. The Role of Pregnancy, Perinatal Factors and Hormones in Maternal Cancer Risk: A Review of the Evidence. J Intern Med (2018) 283(5):430–45. doi: 10.1111/joim.12747 PMC6688839 29476569

[29] Vetter MH Smith B Benedict J Hade EM Bixel K Copeland LJ. Preoperative Predictors of Endometrial Cancer at Time of Hysterectomy for Endometrial Intraepithelial Neoplasia or Complex Atypical Hyperplasia. Am J Obstet Gynecol (2020) 222(1):60.e1–.e7. doi: 10.1016/j.ajog.2019.08.002 PMC7201377 31401259

[30] Wise MR Gill P Lensen S Thompson JM Farquhar CM. Body Mass Index Trumps Age in Decision for Endometrial Biopsy: Cohort Study of Symptomatic Premenopausal Women. Am J Obstet Gynecol (2016) 215(5):598.e1–e8. doi: 10.1016/j.ajog.2016.06.006 27287687

[31] Pintican R Bura V Zerunian M Smith J Addley H Freeman S. MRI of the Endometrium - From Normal Appearances to Rare Pathology. Br J Radiol (2021) 94:20201347. doi: 10.1259/bjr.20201347 34233457 PMC9327760

[32] Levy A Medjhoul A Caramella C Zareski E Berges O Chargari C. Interest of Diffusion-Weighted Echo-Planar MR Imaging and Apparent Diffusion Coefficient Mapping in Gynecological Malignancies: A Review. J Magn Reson Imaging (2011) 33(5):1020–7. doi: 10.1002/jmri.22546 21509857

[33] Zhang Q Peng Y Liu W Bai J Zheng J Yang X. Radiomics Based on Multimodal MRI for the Differential Diagnosis of Benign and Malignant Breast Lesions. J Magn Reson Imaging (2020) 52:596–607. doi: 10.1002/jmri.27098 32061014

[34] Xu M Fang M Zou J Yang S Yu D Zhong L. Using Biparametric MRI Radiomics Signature to Differentiate Between Benign and Malignant Prostate Lesions. Eur J Radiol (2019) 114:38–44. doi: 10.1016/j.ejrad.2019.02.032 31005174

[35] Moharamzad Y Davarpanah AH Yaghobi Joybari A Shahbazi F Esmaeilian Toosi L Kooshkiforooshani M. Diagnostic Performance of Apparent Diffusion Coefficient (ADC) for Differentiating Endometrial Carcinoma From Benign Lesions: A Systematic Review and Meta-Analysis. Abdom Radiol (NY) (2020) 46:1115–28. doi: 10.1007/s00261-020-02734-w 32935258

[36] Chen X Wang X Gan M Li L Chen F Pan J. MRI-Based Radiomics Model for Distinguishing Endometrial Carcinoma From Benign Mimics: A Multicenter Study. Eur J Radiol (2021) 146:110072. doi: 10.1016/j.ejrad.2021.110072 34861530

[37] Yan BC Li Y Ma FH Feng F Sun MH Lin GW. Preoperative Assessment for High-Risk Endometrial Cancer by Developing an MRI- and Clinical-Based Radiomics Nomogram: A Multicenter Study. J Magn Reson Imaging (2020) 52(6):1872–82. doi: 10.1002/jmri.27289 32681608

[38] Kang Y Choi SH Kim YJ Kim KG Sohn CH Kim JH. Gliomas: Histogram Analysis of Apparent Diffusion Coefficient Maps With Standard- or High-B-Value Diffusion-Weighted MR Imaging–Correlation With Tumor Grade. Radiology (2011) 261(3):882–90. doi: 10.1148/radiol.11110686 21969667

[39] Giannella L Delli Carpini G Sopracordevole F Papiccio M Serri M Giorda G. Atypical Endometrial Hyperplasia and Unexpected Cancers at Final Histology: A Study on Endometrial Sampling Methods and Risk Factors. Diag (Basel) (2020) 10(7):474. doi: 10.3390/diagnostics10070474 PMC7400146 32668563

[40] Leitao MM Jr. Han G Lee LX Abu-Rustum NR Brown CL Chi DS. Complex Atypical Hyperplasia of the Uterus: Characteristics and Prediction of Underlying Carcinoma Risk. Am J Obstet Gynecol (2010) 203(4):349.e1–6. doi: 10.1016/j.ajog.2010.05.004 20576254

[41] Fanfani F Monterossi G Di Meo ML La Fera E Dell'Orto F Gioe A. Standard Ultra-Staging Compared to One-Step Nucleic Acid Amplification for the Detection of Sentinel Lymph Node Metastasis in Endometrial Cancer Patients: A Retrospective Cohort Comparison. Int J Gynecol Cancer (2020) 30(3):372–7. doi: 10.1136/ijgc-2019-000937 31996396

[42] Visser NC Sparidaens EM van den Brink JW Breijer MC Boss EA Veersema S. Long-Term Risk of Endometrial Cancer Following Postmenopausal Bleeding and Reassuring Endometrial Biopsy. Acta Obstet Gynecol Scand (2016) 95(12):1418–24. doi: 10.1111/aogs.13022 PMC5132156 27633936

[43] Ozdemir S Celik C Gezginc K Kiresi D Esen H. Evaluation of Endometrial Thickness With Transvaginal Ultrasonography and Histopathology in Premenopausal Women With Abnormal Vaginal Bleeding. Arch Gynecol Obstet (2010) 282(4):395–9. doi: 10.1007/s00404-009-1290-y 19921229

