# Supplementary material for: Multimodal MRI-Based Radiomics-Clinical Model for Preoperatively Differentiating Concurrent Endometrial Carcinoma From Atypical Endometrial Hyperplasia
Source: Front Oncol. 2022 May 27;12:887546. doi: 10.3389/fonc.2022.887546 (PMC9186045; doi:10.3389/fonc.2022.887546)
Supplement: Supplementary file 1 [file DataSheet_1.pdf]

Supplementary Materials

Table S1. Detailed radiomics features

Categories	Number	Features
Shape-based features	14	Mesh Volume; Voxel Volume; Surface Area; Surface Area to Volume ratio; Sphericity; Maximum 3D diameter; Maximum 2D diameter (Slice); Maximum 2D diameter (Column); Maximum 2D diameter (Row); Major Axis Length; Minor Axis Length; Least Axis Length; Elongation; Flatness
First-order features	18	Energy; Total Energy; Entropy; Minimum; 10th percentile; 90th percentile; Maximum; Mean; Median; Interquartile Range; Range; Mean Absolute Deviation (MAD); Robust Mean Absolute Deviation (rMAD); Root Mean Squared (RMS); Skewness; Kurtosis; Variance; Uniformity
Gray level co-occurrence matrix (GLCM) features	22	Autocorrelation; Joint Average; Cluster Prominence; Cluster Shade; Cluster Tendency; Contrast; Correlation; Difference Average; Difference Entropy; Difference Variance; Joint Energy; Joint Entropy; Informational Measure of Correlation (IMC) 1; Informational Measure of Correlation (IMC) 2; Inverse Difference Moment (IDM); Inverse Difference Moment Normalized (IDMN); Inverse Difference (ID); Inverse Difference Normalized (IDN); Inverse Variance; Maximum Probability; Sum Entropy; Sum of Squares
Gray level size zone matrix (GLSZM) features	16	Small Area Emphasis (SAE); Large Area Emphasis (LAE); Gray Level Non-Uniformity (GLN); Gray Level Non-Uniformity Normalized (GLNN); Size-Zone Non-Uniformity (SZN); Size-Zone Non-Uniformity Normalized (SZNN); Zone Percentage (ZP); Gray Level Variance (GLV); Zone Variance (ZV); Zone Entropy (ZE); Low Gray Level Zone Emphasis (LGLZE); High Gray Level Zone Emphasis (HGLZE); Small Area Low Gray Level Emphasis (SALGLE); Small Area High Gray Level Emphasis (SAHGLE); Large Area Low Gray

		Level Emphasis (LALGLE); Large Area High Gray Level Emphasis (LAHGLE)
Gray level run length matrix (GLRLM) features	16	Short Run Emphasis (SRE); Long Run Emphasis (LRE); Gray Level Non-Uniformity (GLN); Gray Level Non-Uniformity Normalized (GLNN); Run Length Non-Uniformity (RLN); Run Length Non-Uniformity Normalized (RLNN); Run Percentage (RP); Gray Level Variance (GLV); Run Variance (RV); Run Entropy (RE); Low Gray Level Run Emphasis (LGLRE); High Gray Level Run Emphasis (HGLRE); Short Run Low Gray Level Emphasis (SRLGLE); Short Run High Gray Level Emphasis (SRHGLE); Long Run Low Gray Level Emphasis (LRLGLE); Long Run High Gray Level Emphasis (LRHGLE)
Gray level dependence matrix (GLDM) features	14	Small Dependence Emphasis (SDE); Large Dependence Emphasis (LDE); Gray Level Non-Uniformity (GLN); Dependence Non-Uniformity (DN); Dependence Non-Uniformity Normalized (DNN); Gray Level Variance (GLV); Dependence Variance (DV); Dependence Entropy (DE); Low Gray Level Emphasis (LGLE); High Gray Level Emphasis (HGLE); Small Dependence Low Gray Level Emphasis (SDLGLE); Small Dependence High Gray Level Emphasis (SDHGLE); Large Dependence Low Gray Level Emphasis (LDLGLE); Large Dependence High Gray Level Emphasis (LDHGLE)

Table S2. Endometrial thickness (ET) in the subgroups of AEH and CEC patients.

Subgroups	Training Set (n=87)		Validation Set (n=35)		Total (n=122)		*p* value
	AEH (n=57)	CEC (n=30)	AEH (n=21)	CEC (n=14)	AEH (n=78)	CEC (n=44)	
**Premenopausal**†							0.006*
ET ≤ 11mm	28 (62.2)	8 (30.8)	10 (66.7)	4 (44.4)	38 (63.3)	12 (34.3)	
ET > 11mm	17 (37.8)	18 (69.2)	5 (33.3)	5 (55.6)	22 (36.7)	23 (65.7)	
**Postmenopausal**†							0.219
ET ≤ 11mm	9 (75.0)	2 (50.0)	4 (66.7)	2 (40.0)	13 (72.2)	4 (44.4)	
ET > 11mm	3 (25.0)	2 (50.0)	2 (33.3)	3 (60.0)	5 (27.8)	5 (55.6)	
**Parous**†							0.008*
ET ≤ 11mm	35 (63.6)	8 (32.0)	14 (70.0)	6 (54.5)	49 (65.3)	14 (38.9)	
ET > 11mm	20 (36.4)	17 (68.0)	6 (30.0)	5 (45.5)	26 (34.7)	22 (61.1)	
**Nulliparous**†							0.491
ET ≤ 11mm	2 (100.0)	2 (40.0)	0 (0.0)	0 (0.0)	2 (66.7)	2 (25.0)	
ET > 11mm	0 (0.0)	3 (60.0)	1 (100.0)	3 (100.0)	1 (33.3)	6 (75.0)	

† Data in parentheses are percentages.

* $p < 0.05$.

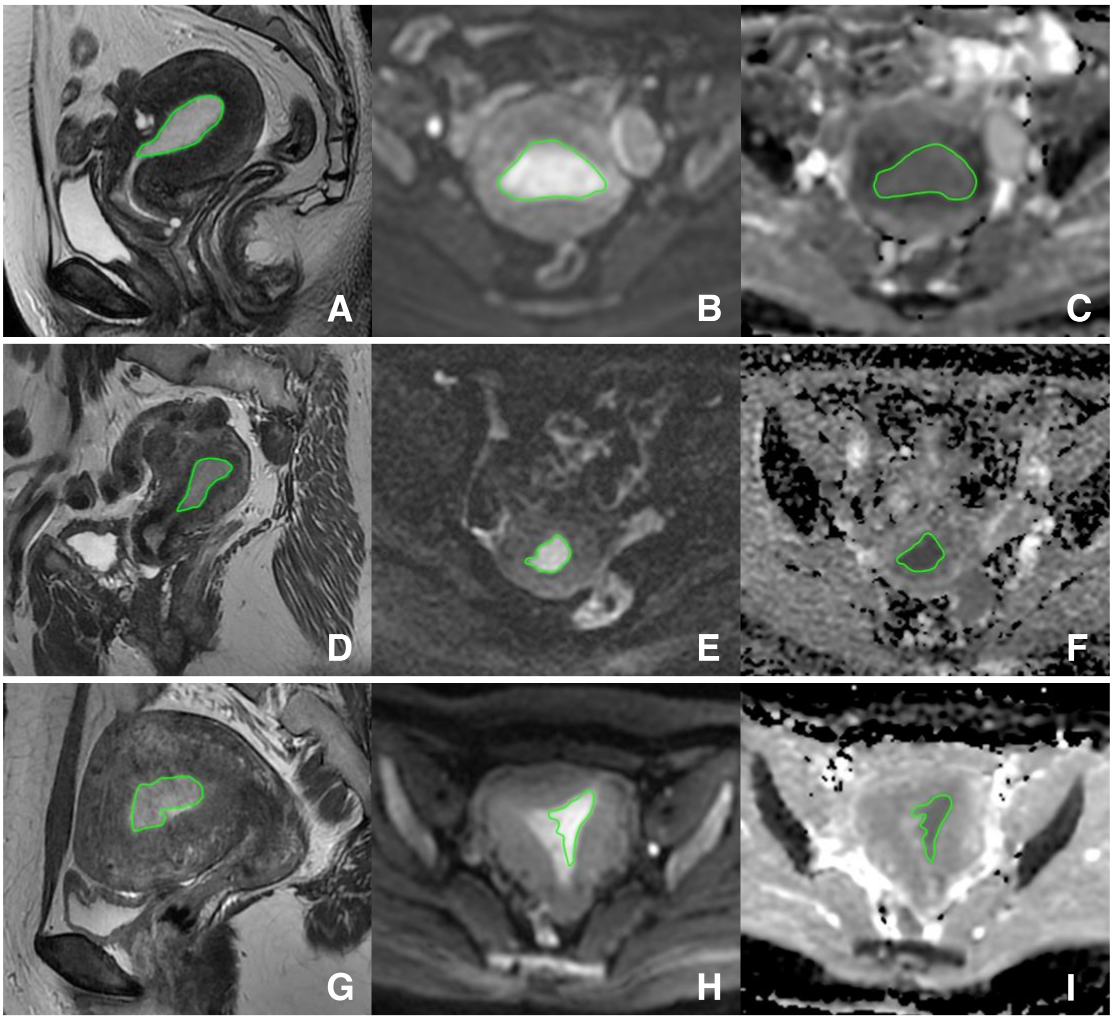

Figure.S1 Findings of three patients with surgically proven AEH and stage IA CECs. Sagittal T2WI (A, D, G), axial DWI (b=800s/mm2; B, E, H), and axial ADC maps (C, F, I). (A-C) The AEH lesion showed similar signal intensity with normal endometrium on T2WI, DWI, and ADC map. We contoured the entire endometrium as the VOI. (D-F) The CEC lesion also showed similar signal intensity with adjacent endometrium on T2WI, DWI, and ADC map. We contoured the entire endometrium as the VOI. (G-I) The CEC lesion was presented with slightly lower signal intensity on T2WI, higher signal intensity on DWI and a lower value on the ADC map compared with adjacent endometrium. We contoured the visible tumor as the VOI.

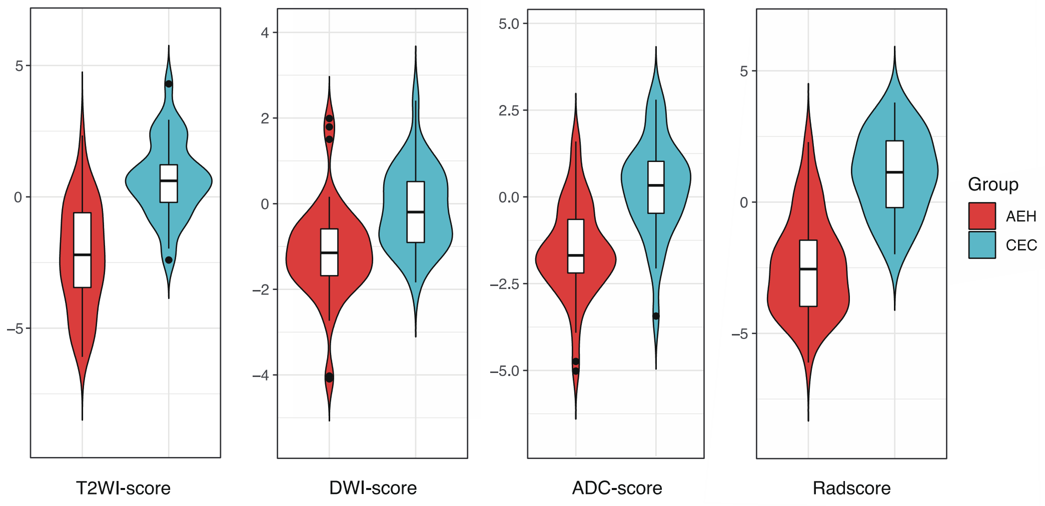

Figure.S2 Distribution of the radiomic scores (T2WI-score, DWI-score, ADC-score and Radscore) between AEH and CEC groups in the training set (T2WI-score [median, -2.21 vs 0.61, p<0.001], DWI-score [median, -1.14 vs -0.19, p<0.001], ADC-score [median, -1.69 vs 0.33, p<0.001], and Radscore [median, -2.55 vs 1.13, p<0.001]). Density width indicates the frequency of the radiomic scores; middle line in each box indicates the median value of the radiomic scores, and lower and upper boundaries of the box indicate the first and third quartiles, respectively. Whiskers indicate the 95% confidence interval, and dots show outliers.

AEH, atypical endometrial hyperplasia*; CEC*, concurrent endometrial cancer with AEH*; T2WI*, T2-weighted imaging

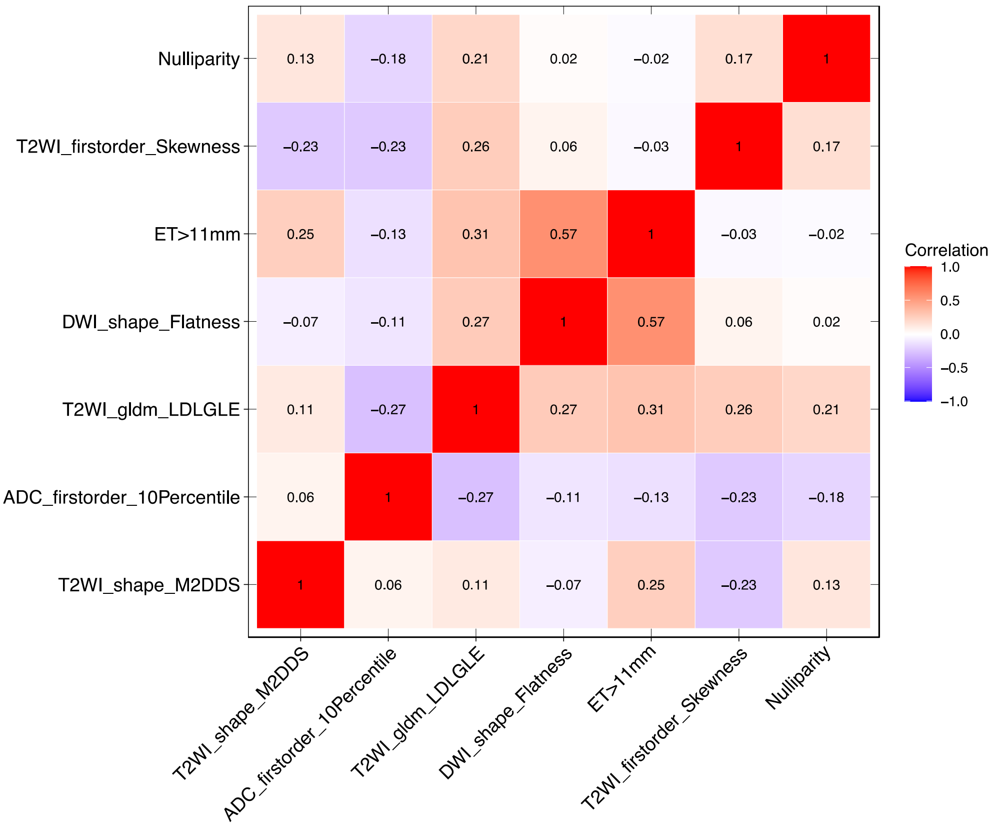

Figure.S3 The correlation heatmap shows auto- and cross-correlation of selected features in the radiomics-clinical model derived from the training set. Significant cross-correlation (Pearson correlation coefficient $r > 0.7$) was not observed among features, which indicated that our collinearity analysis worked well for dimension reduction.

ET, endometrial thickness*; LDLGLE*, Large Dependence Low Gray Level Emphasis*; M2DDS*, Maximum 2D Diameter Slice

Method S1. The formula generated from the logistic regression model. The radiomics score was defined as a linear combination of the selected features weighted by their respective coefficient. The mathematical formula of the radiomics score was defined as follows:

$$y = \sum_{j}^{n} \beta_j x_j + \beta_0$$

where n is the number of selected features, x_j (j = 1, 2, ..., n) is the selected features, and β_j (j = 1, 2, ..., n) is the coefficients, while β_0 is the intercept.

The logistic regression model was defined as follows:

$$f(y) = \frac{1}{1 + e^{-y}}$$

where y is the radiomics scores.

Formula in the radiomics signatures.

1）**T2WI-score** = -19.029 × glszm_SizeZoneNonUniformityNormalized +10.231 × glszm_SmallAreaLowGrayLevelEmphasis + 0.00102× firstorder_10Percentile + -0.069 × shape_Maximum2DDiameterSlice + 7.225 × shape_Flatness + 1.968 × firstorder_Skewness + 0.143 × gldm_LargeDependenceLowGrayLevelEmphasis + 1.457

2）**DWI-score** = -0.029 × shape_Maximum2DDiameterRow + -1.579 × firstorder_Kurtosis + 6.175 × shape_Flatness + 2.650

3）**ADC-score** = -0.00926 × firstorder_10Percentile + 8.480

4）**Radscore** = -0.00609 × T2WI_shape_Maximum2DDiameterSlice + 0.178 × T2WI_gldm_LargeDependenceLowGrayLevelEmphasis + 4.815 × DWI_shape_Flatness + 1.046 × T2WI_firstorder_Skewness + -0.00855 × ADC_firstorder_10Percentile + 7.544

Formula in the radiomics-clinical model.

Clinical-radiomic (nomogram) score = 0.980 × Radscore + 1.609 × (Nulliparity=n_1) + 1.258 × (ET=n_2) + -0.854

Where

Nulliparity = n_1 is when the status of nulliparity is yes， n_1=1; Otherwise, n_1=0

ET = n_2 is when the endometrial thickness > 11mm, n_2=1; Otherwise, n_2=0
